# Human Tissue Angiotensin Converting Enzyme (ACE) Activity Is Regulated by Genetic Polymorphisms, Posttranslational Modifications, Endogenous Inhibitors and Secretion in the Serum, Lungs and Heart

**DOI:** 10.3390/cells10071708

**Published:** 2021-07-06

**Authors:** Viktor Bánhegyi, Attila Enyedi, Gábor Áron Fülöp, Attila Oláh, Ivetta Mányiné Siket, Csongor Váradi, Klaudia Bottyán, Mária Lódi, Alexandra Csongrádi, Azeem J. Umar, Miklós Fagyas, Dániel Czuriga, István Édes, Miklós Pólos, Béla Merkely, Zoltán Csanádi, Zoltán Papp, Gábor Szabó, Tamás Radovits, István Takács, Attila Tóth

**Affiliations:** 1 Division of Clinical Physiology, Department of Cardiology, Faculty of Medicine, University of Debrecen, 4032 Debrecen, Hungary; viktor.banhegyi@uk-halle.de (V.B.); fulop.gabor.aron@gmail.com (G.Á.F.); sivi@med.unideb.hu (I.M.S.); klaudia.bottyan.95@gmail.com (K.B.); lodrin91@gmail.com (M.L.); alexandra.csongradi@gmail.com (A.C.); azeemjumar@gmail.com (A.J.U.); fagyasmiklos@med.unideb.hu (M.F.); pappz@med.unideb.hu (Z.P.); 2 Doctoral School of Kálmán Laki, University of Debrecen, 4032 Debrecen, Hungary; 3 Department of Cardiac Surgery, University of Halle, 06120 Halle (Saale), Germany; gabor.szabo@uk-halle.de; 4 Division of Thoracic Surgery, Department of Surgery, Faculty of Medicine, University of Debrecen, 4032 Debrecen, Hungary; drenyediattila@gmail.com (A.E.); varadi.csongor@med.unideb.hu (C.V.); takacsistvan63@gmail.com (I.T.); 5 Heart and Vascular Center, Semmelweis University, 1122 Budapest, Hungary; o.attilio@gmail.com (A.O.); polos.miklos@med.semmelweis-univ.hu (M.P.); merkely.bela@kardio.sote.hu (B.M.); radovitstamas@yahoo.com (T.R.); 6 Division of Cardiology, Department of Cardiology, Faculty of Medicine, University of Debrecen, 4032 Debrecen, Hungary; dczuriga@med.unideb.hu (D.C.); edes@med.unideb.hu (I.É.); csanadi.zoltan@med.unideb.hu (Z.C.); 7 Vascular Biology and Myocardial Pathophysiology Research Group, Hungarian Academy of Sciences—University of Debrecen, 4032 Debrecen, Hungary

**Keywords:** angiotensin converting enzyme, ACE, cardiovascular disease, lung, heart, tissue-localized, circulating

## Abstract

**Objective:** Inhibitors of the angiotensin converting enzyme (ACE) are the primarily chosen drugs to treat heart failure and hypertension. Moreover, an imbalance in tissue ACE/ACE2 activity is implicated in COVID-19. In the present study, we tested the relationships between circulating and tissue (lung and heart) ACE levels in men. **Methods:** Serum, lung (*n* = 91) and heart (*n* = 72) tissue samples were collected from Caucasian patients undergoing lung surgery or heart transplantation. ACE I/D genotype, ACE concentration and ACE activity were determined from serum and tissue samples. Clinical parameters were also recorded. **Results:** A protocol for ACE extraction was developed for tissue ACE measurements. Extraction of tissue-localized ACE was optimal in a 0.3% Triton-X-100 containing buffer, resulting in 260 ± 12% higher ACE activity over detergent-free conditions. SDS or higher Triton-X-100 concentrations inhibited the ACE activity. Serum ACE concentration correlated with ACE I/D genotype (II: 166 ± 143 ng/mL, *n* = 19, ID: 198 ± 113 ng/mL, *n* = 44 and DD: 258 ± 109 ng/mL, *n* = 28, *p* < 0.05) as expected. In contrast, ACE expression levels in the lung tissue were approximately the same irrespective of the ACE I/D genotype (II: 1423 ± 1276 ng/mg, ID: 1040 ± 712 ng/mg and DD: 930 ± 1273 ng/mg, *p* > 0.05) in the same patients (values are in median ± IQR). Moreover, no correlations were found between circulating and lung tissue ACE concentrations and activities (Spearman’s *p* > 0.05). In contrast, a significant correlation was identified between ACE activities in serum and heart tissues (Spearman’s Rho = 0.32, *p* < 0.01). Finally, ACE activities in lung and the serum were endogenously inhibited to similar degrees (i.e., to 69 ± 1% and 53 ± 2%, respectively). **Conclusion:** Our data suggest that circulating ACE activity correlates with left ventricular ACE, but not with lung ACE in human. More specifically, ACE activity is tightly coordinated by genotype-dependent expression, endogenous inhibition and secretion mechanisms.

## 1. Introduction

The renin-angiotensin-aldosterone system (RAAS) plays a crucial role in the fluid and salt homeostasis. One of the key biochemical steps within the RAAS is conversion of the inactive angiotensin I decapeptide (AngI) to active angiotensin II (AngII) octapeptide by angiotensin converting enzyme (ACE). ACE was first identified in 1956 by Skeggs et al. [1], and ACE inhibitors were subsequently introduced in clinical practice. They represent a first line therapy for a wide range of cardiovascular maladies, including hypertension [2,3] and heart failure [4]. It is important to note that AngII generation by ACE is reversed by its isoform ACE2 (which eliminates AngII). Therefore, the physiological level of AngII is usually determined by the balance between ACE and ACE2 activities in tissues. This balance is important in cardiovascular diseases [5,6,7], and also in COVID-19. Regarding the latter, ACE2 is the cellular receptor for the SARS-CoV-2 [8] and it is proposed that some symptoms of COVID-19 are mediated by disrupted ACE/ACE2 balance [9,10].

The molecular properties of a successful ACE inhibitor generally include low lipophilicity (with the exception of fosinopril) [11]. This indicates that the primary target of these drugs is the water-soluble (circulating) form of the enzyme. Accordingly, factors affecting circulating ACE activities have been implicated in the pathomechanism of cardiovascular disease. Circulating ACE concentration is controlled by a genetic polymorphism of the ACE gene (an insertion/deletion polymorphism, I/D polymorphism) [12], being implicated in systolic heart failure [13].

According to a widely accepted consensus, ACE is expressed primarily by endothelial cells, particularly those of the lung [14], and subsequently released into the circulation. However, the human heart also expresses ACE [15], suggesting that the lung is probably not the only organ contributing to circulating ACE in humans. Moreover, levels of ACE expressions in kidneys and in small intestines were also found to be comparable to those in the lung [16].

Another important finding was the identification of an endogenous inhibitor for circulating ACE [17], which was later identified as serum albumin [18]. Serum albumin almost fully inhibits circulating ACE activity at its physiological concentrations [18]. Accordingly, ACE activity is localized to the tissues (where albumin concentration is low), suggesting that ACE inhibitory drugs are acting on tissue-localized ACE. Tissue ACE/ACE2 balance (tissue AngII production) can be modulated by expression (affected by polymorphisms of both ACE and ACE2), by shedding and potentially by interacting proteins (endogenous inhibition, similarly to albumin in the serum). These factors implicate a potentially complex interplay between tissue ACE/ACE2 expression and circulating ACE/ACE2 activity in contexts of both cardiovascular disease and COVID-19.

In the present study, we tested the links between ACE activity and its genotype-specific expression, endogenous inhibitors and ACE secretion in clinical samples. ACE activity and ACE concentration were measured in human sera and tissue (lung and heart) samples obtained from patients undergoing lung surgery or heart transplantation. Circulating, but not lung tissue ACE expression was regulated by ACE I/D genetic polymorphism. In contrast, both circulating and lung tissue ACE activities were regulated by endogenous inhibition. Finally, there was a correlation between circulating and left ventricular ACE activity, but not between circulating and lung ACE activity/expression, suggestive for cardiac-specific secretion mechanisms contributing to circulating ACE activity.

## 2. Materials and Methods

### 2.1. Patients

This prospective study was done involving patients with lung surgeries at the clinical ward of the University of Debrecen and patients undergoing heart transplantation at the Heart and Vascular Center at Semmelweis University, Budapest. The study was authorized by the Medical Research Council of Hungary (20753-7/2018/EÜIG for patients undergoing lung surgery and ETT TUKEB 7891/2012/EKU (119/PI/12.) for patients with heart transplantation). Tissue and blood samples were obtained from patients undergoing thoracic-surgical interventions (lung samples) or heart transplantation (pseudonymized explanted heart samples from the left ventricular anterior wall and blood plasma samples were obtained from the Transplantation Biobank of the Heart and Vascular Center at Semmelweis University, Budapest, Hungary). All enrolled patients gave their individual informed consents according to the Declaration of Helsinki.

Native blood samples were aliquoted for DNA isolation and subsequently frozen, or incubated at room temperature for 60 min and centrifuged at 1500× *g* for 15 min. The obtained sera fractions and tissue samples were stored at −70 °C until the biochemical measurements were performed. Case history, medication, comorbidities and basic cardiovascular parameters were recorded in agreement with the General Data Protection Regulation (EU GDPR 2016/679) of the European Parliament and Council. Selected patient characteristics are summarized in Table 1.

### 2.2. ACE I/D Genotype Determination

Patient’s DNA was extracted from peripheral blood using a commercial DNA extraction kit (FlexiGene; Qiagen GmbH, Hilden, Germany). DNA fragments were amplified with polymerase chain reaction primers (forward: CTGGAGACCACTCCCACTCTTTCT and reverse: GATGTGGCCATCACATTCGTCAGAT), as done before in the laboratory [18]. After amplification, PCR products were separated using electrophoresis on 3% polyacrylamide gels and genotypes (II, ID, DD) were identified by SybrSafe staining.

### 2.3. Tissue Processing for ACE Activity and Expression Measurements

Human lung and left ventricular heart tissue samples were mechanically crushed in liquid nitrogen with a pestle and mortar. Five ml of 100 mM TRIS-HCl, pH 7.0 was then added to each g of tissue (wet weight) on ice. Samples were homogenized with a tissue homogenizer (Bio-Gen PRO200, PRO Scientific, Oxford, CT, USA) and centrifuged at 16,100× *g* for 5 min. Supernatants were collected and kept frozen until biochemical determinations.

### 2.4. Procedures to Measure the Effects of Detergents

In many cases, effects of detergents were also tested on the ACE extraction from the lung tissue. In these experiments, homogenization buffer (100 mM TRIS-HCl, pH 7.0) was supplemented with the indicated concentration of Triton-X-100, Triton-X-114 and SDS.

### 2.5. ACE Activity Measurements

Tissue and circulating ACE activity measurements were performed as described before [19]. In short, cleavage of the quenched fluorescent substrate (Abz-FRK(Dnp)P-OH was used to measure the activity in a kinetic assay. The measurement mixture contained 100 mM TRIS-HCl, pH 7.0, 50 mM NaCl, 10 µM ZnCl_2_, 10 µM Abz-FRK(Dnp)P-OH and the intended amount of sera/tissue sample, in addition to the detergents mentioned above. ACE activity was measured at 37 °C. ACE activity measurements were performed with a plate reader at **λ**_ex_ 340 nm and **λ**_em_ 405 nm (NovoStar, BMG Labtech, Ortenberg, Germany). Results were accepted when the goodness of the fit (*r*^2^) was at least 0.90. The ACE activity was calculated based on the rate of the observed increase in fluorescent intensity (AU/min), which was transformed to absolute units based on a calibration curve with the Abz fluorophore.

### 2.6. ACE Concentration Measurements

ACE expression was measured by an Enzyme-Linked Immunosorbent Assay (Catalog No. DY929; R&D Systems, Minneapolis, MN, USA) according to the manufacturer’s instructions. In short, the capture antibody was diluted to working concentration of 80 ng/well in Dulbecco’s phosphate-buffer saline (DPBS) at room temperature. The remaining binding sites were blocked with 10 mg/mL bovine serum albumin dissolved in DPBS. Human serum/lung samples were diluted 100-fold in the same buffer (10 mg/mL of bovine serum albumin in DPBS) and incubated with the immobilized primary antibodies for 2 h. Capture antibody-bound ACE was labeled using a biotinylated detection antibody, 20 ng/well for 2 h. Streptavidin-conjugated horseradish-peroxidase (200-fold-diluted stock from the kit) was added to the wells and incubated for 30 min. Immunocomplexes were detected with a chromogenic substrate solution containing 0.3 mg/mL TMB (3,3′,5,5′-tetramethylbenzidine), 0.1 mM H_2_O_2_ and 50 mM acetic acid (incubation time was approximately 30 min). The reaction was terminated by the addition of 0.5 M HCl and was evaluated by measuring the absorbance at 450 nm. ACE concentration was calculated using a calibration curve. Serum ACE concentration was given as ng/mL of serum.

### 2.7. Chemicals

All chemicals were from Sigma-Aldrich (St. Louis, MO, USA) if not indicated otherwise.

### 2.8. Statistical Analysis

Normality check was performed by Kolmogorov–Smirnoff test. An ANOVA was applied for values showing normal distribution. Mann–Whitney or Kruskal–Wallis tests were used as non-parametric tests with Dunn’s multiple comparison post hoc test. A *χ*^2^ test was performed to compare the different clinical and genotype subgroups. The differences were considered to be significant when *p* < 0.05. Statistical analyses were performed by GraphPad Prism, version 5.0 (GraphPad Software, Inc., San Diego, CA, USA).

## 3. Results

### 3.1. Results

#### 3.1.1. Development of a Protocol for Tissue ACE Extraction

**Figure 1:**
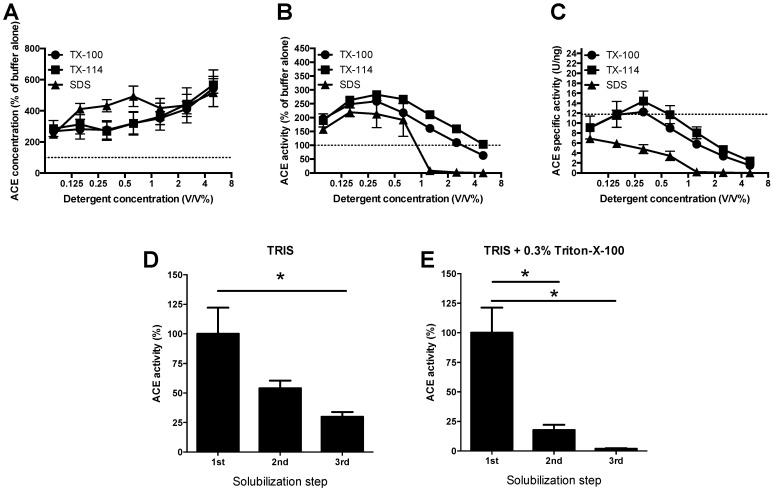
ACE extraction method—A role for detergents. Human lung tissue samples were homogenized in SDS, Triton-X-100 and Triton-X-114 containing buffers. Final concentrations in activity measurements are shown on the x-axes (note, homogenates were two-fold diluted in the activity measurement mixture). The homogenates were centrifuged and the supernatants were collected, diluted to 1 mg/mL (with the same buffer, containing the indicated detergents). ACE concentration was measured by ELISA (**A**), while activity was measured by a kinetic assay (**B**). The ratio of the activity and concentration values yielded the specific activity, which is shown in (**C**). In some cases, the homogenization procedure was repeated using the ACE depleted pellets after homogenization. The number of these repeated extraction cycles is indicated in panels (**D**,**E**). In these cases, the ACE activity was expressed as the percentage of the first supernatants ((**D**): buffer without detergent, (**E**): Buffer with 0.3% Triton-X-100). Symbols (**A**–**C**) and bars (**D**,**E**) represent the mean, error bars show the S.E.M. of *n* = 5–9. Significant differences (*p* < 0.05) are labelled by the asterisks.

We aimed to compare circulating and tissue ACE activities and expressions. First, we developed a suitable tissue extraction protocol for the lung. Detergents (Triton-X-100, Triton-X-114 and SDS) were tested in the range of 0.06–5.0 *v*/*v*%. Application of these detergents increased the yield to approximately 250% ACE concentration even at their lowest (0.06%) concentrations (Figure 1A), when compared to the buffer without detergents (100%, represented by the dotted line, Figure 1A). The increase in ACE solubilization was approximately 550% at the highest (5.0 *v*/*v*%) detergent concentrations (Figure 1A). Nonetheless, this gradual increase in solubilized ACE concentration was only partially paralleled by the ACE activity. The activity increased at the lower detergent concentrations, reaching the maximum at 0.3 *v*/*v*% (at approximately 250%, Figure 1B) and declined at higher concentrations. Calculated specific activities suggested inhibition of the ACE activity by the detergents. SDS inhibited ACE even at the lowest tested concentrations, while the Triton based detergents inhibited the enzyme activity at concentrations of 0.6% and higher (Figure 1C).

Solubilized ACE was collected in the supernatant and the pellets were re-processed two additional times (same protocol than that for initial tissue ACE extraction) to see how much ACE remained in the processed tissue. Significant ACE activity remained in the pellets without detergents, suggestive for incomplete ACE extraction (Figure 1D). ACE extraction was significantly improved in the presence of Triton-X-100 (at 0.3 *v*/*v*%) (Figure 1E).

#### 3.1.2. ACE I/D Polymorphism as a Quantitative Trait Locus for ACE Expression in the Blood, but Not in the Lung

**Figure 2:**
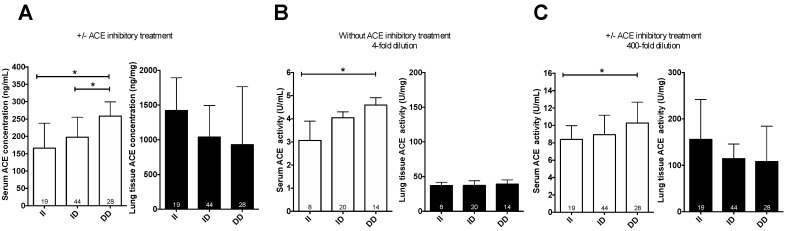
Missing effect of genotype on lung tissue ACE activity and expression. The ACE concentration was determined by ELISA in sera and lung tissue samples of patients (**A**). The label “+/− ACE inhibitory treatment” means that patients with ACEi medication were included in the analysis. ACE activity in patients without ACE inhibitory medication at low dilution levels is shown in (**B**). ACE activity was also plotted at sufficiently high dilution levels (400-fold), where the ACE inhibitory drug had a negligible effect, to include all patients (**C**). Empty bars show serum concentrations (**A**) or activities (**B**,**C**), while filled bars represent lung tissue concentrations (**A**) and activities (**B**,**C**). Bars show the median, error bars represent IQR. Numbers within the bars indicate the number of biological replicates (patient samples) in the groups. Genotypes are indicated below the bars. Significant differences (*p* < 0.05) are shown by the brackets and asterisks.

Serum ACE concentration was influenced by ACE I/D polymorphism in patients with pulmonary surgery (Figure 2A). Patients with II, ID and DD genotype had 166 ± 143 ng/mL, 198 ± 113 ng/mL and 258 ± 109 ng/mL circulating ACE concentrations, respectively, suggestive for a dominance of the D allele in determining circulating ACE concentration (values are in median ± IQR). In contrast, there was no effect of ACE I/D polymorphism on ACE expression in lungs of the same patients (tissue ACE concentration was 1423 ± 1276 ng/mg, 1040 ± 712 ng/mg and 930 ± 1273 ng/mg, respectively). ACE D genotype resulted in elevated circulating (serum) ACE activities in patients without ACE inhibitory medications (3.1 ± 1.4 U/mL, 4.0 ± 1.4 U/mL and 5.0 ± 2.5 U/mL, Figure 2B). A similar correlation was found in all patients (irrespective to ACE inhibitory medication) when the dilution level was high enough to neutralize the effects medications and endogenous ACE inhibition (8.4 ± 4.9 U/mL, 8.9 ± 4.2 U/mL and 10.3 ± 3.9 U/mL, Figure 2C). In contrast, there were no apparent links between ACE I/D genotype and lung ACE activities in the same patients (37 ± 18 U/mg, 37 ± 18 U/mg and 39 ± 15 U/mg, Figure 2B and 156 ± 161 U/mg, 115 ± 68 U/mg and 108 ± 121 U/mg, Figure 2C). Note that the higher activities at higher dilution levels (Figure 2B vs. Figure 2C) illustrate the presence of endogenous inhibitors affecting enzyme activity at physiological (undiluted) conditions.

#### 3.1.3. Missing Correlation between Serum and Lung Tissue ACE Expression/Activity

**Figure 3:**
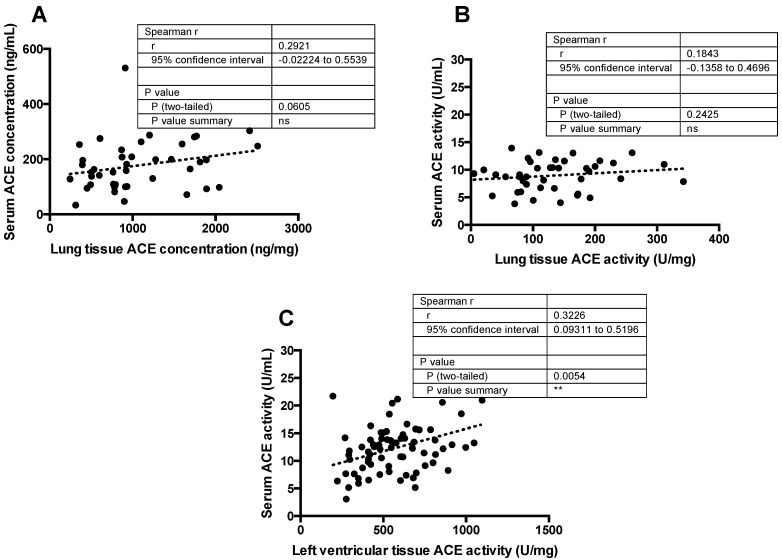
Correlation between circulating and tissue ACE levels in the same patient samples. Serum ACE concentration or activity was plotted as the function of the ACE concentration (**A**) or activity (**B**) in lung tissue homogenates or ACE activity in the left ventricle of explanted human heart tissue samples (**C**). The parameters of Spearman’s correlation are shown in the table inserts (“ns” means no significant correlation, asterisks represent the presence of a statistically significant correlation). Each symbol represents the value determined for an individual patient. The linear fits represented by the dotted lines in the graphs show the trendlines.

Surprisingly, no significant correlation was found between ACE concentrations in lung tissue and circulation (Figure 3A). Similarly, there was no correlation between ACE activities in lung tissue and in blood (Figure 3B). In contrast, circulating and cardiac (left ventricular) ACE activities significantly correlated with each other (Figure 3C).

#### 3.1.4. ACE Expression and Activity Are Correlated in Serum and Lung Tissue

**Figure 4:**
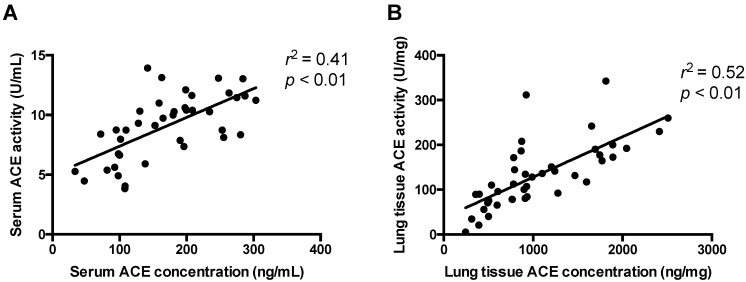
There is a positive correlation between the activity and concentration in the same samples. ACE activity and concentration was measured in the same serum (**A**) and tissue (**B**) samples. Activity was plotted as a function of the concentration. A linear regression was used to establish the correlation between activity and expression. The goodness of fit is represented by the *r*^2^ values, while significance of the correlations by *p* values. Each symbol represents individual values. Linear fits are shown by the line in the graphs.

The missing correlation between ACE levels of lung tissues and sera can, hypothetically, be explained by methodological errors. To rule out methodical inaccuracies ACE activities were plotted as a function of the ACE concentration from the same sources (sera for the circulating ACE and lung tissue homogenate for tissue ACE, Figure 4). A positive linear correlation was found for sera (Figure 4A) and lung tissues (Figure 4B). The observed linear relationships (significant differences from the horizontal lines as characterized by the low *p* values and acceptable fits as shown by the high *r*^2^ values) indicated that the measurements were sufficiently accurate for both sera and lung tissue samples.

#### 3.1.5. ACE Activity Is Regulated by Endogenous Inhibition in the Lung

**Figure 5:**
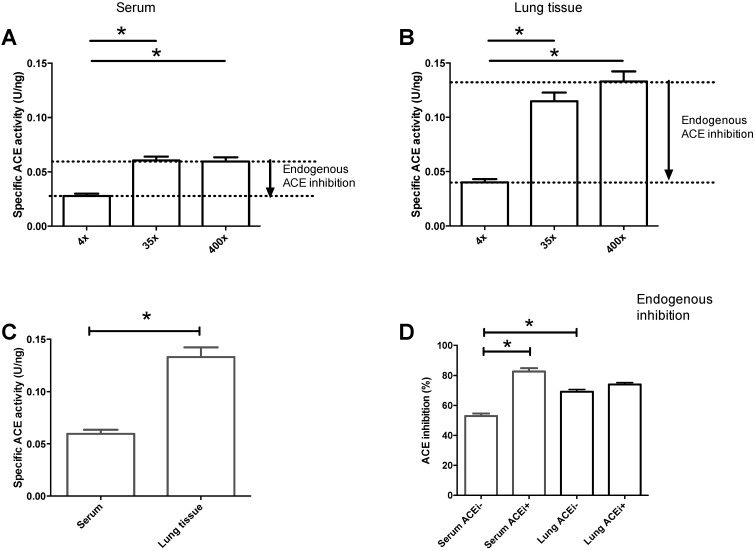
Both circulating and tissue ACE are endogenously inhibited. ACE activities were determined at various dilution levels (4-, 35- and 400-fold dilutions) in sera (**A**) and lung (**B**) homogenates. Activities were normalized to the ACE concentration of the same sample to yield specific enzyme activities, which are plotted on the graphs. Lower specific activity values at lower dilutions suggested endogenous inhibition (indicated by the arrows in (**A**,**B**). Specific activities determined at 400-fold dilutions are shown in (**C**). Levels of inhibition in patients without (ACEi−) or with (ACEi+) ACE inhibitory medications (ACEi) are shown in (**D**). Bars represent the mean, while error bars show the S.E.M. of *n* = 42–47 determinations. Significant differences (*p* < 0.05) are shown by the brackets and asterisks.

We reported earlier that an endogenous inhibitor controls circulating ACE activities [18,20]. In the present study, we paralleled an estimation for this ACE inhibitory effect in the circulation (serum) and lung tissue (Figure 5). Endogenous inhibition was confirmed by the lower apparent activities at low dilutions compared to those at high dilutions for both serum (Figure 5A) and lung tissue (Figure 5B) samples. In addition to this endogenous regulation of ACE activities, there was an apparent difference in the uninhibited (determined at the highest dilutions) specific activities. The specific activity for serum ACE was 0.06 ± 0.004 U/ng, which was approximately half of that for lung tissue ACE (0.13 ± 0.009 U/ng, *p* < 0.05, Figure 5C). The level of endogenous ACE inhibition in sera was 53 ± 2% in patients without ACE inhibitory medication (Figure 5D). The level of inhibition increased to 83 ± 2% (*p* < 0.05, Figure 5D) in patients with ACE inhibitory medication, indicating efficient medical (drug) treatment. The level of endogenous inhibition in lung tissue samples (69 ± 1%, Figure 5D) was higher than that for circulating ACE. The effect of ACE inhibitory medication was absent in lung samples (level of inhibition was 74 ± 1% in patients with ACE inhibitory medication, and no significant difference was observed when it was compared to lung samples without ACE inhibitory medications, Figure 5D).

#### 3.1.6. Tissue-Localized ACE Activity Did Not Correlate with Age or Sex

There was no statistical difference in tissue-localized ACE activity in patients younger or older than 60 years, nor in females or males (Table 2).

## 4. Discussion

It is a widely accepted that angiotensin converting enzyme (ACE) is primarily expressed in endothelial cells [21]. It is also believed that the primary source of circulating ACE is the lung, based on the observation that all lung capillaries express ACE, while ACE expression is only approximately 20% of that in other organs [22]. It was estimated that ~75% of blood ACE originates from lung capillaries [23]. However, it was also found that additional organs, such as small intestines and kidneys, have comparable ACE expression levels to that in the lungs [24]; moreover, the conversion of angiotensin I into angiotensin II (the physiological function of ACE) is extremely high in the human heart when compared to dog, rabbit and mouse hearts [25].

ACE insertion/deletion (ACE I/D) genotype was suggested to be a genetic trait locus determining circulating ACE levels [12] and was associated with various cardiovascular diseases, including heart failure [13]. This suggests that the organ which provides the circulating ACE must have an ACE I/D genotype-dependent expression pattern. Indeed, tissue ACE levels showed a correlation with the genetic background in the human heart [15]. It is important to note that ACE expression is not only regulated by the genetic background, but also by physiological factors, such as redox state. In particular, it appears that ACE expression is inhibited by NO and facilitated by NOS inhibition [26]. It suggests that ACE expression is also regulated by endothelial function not only passively by the number of endothelial cells. In general, the biochemical milieu is the driver of ACE enzymatic activity, acting on the cells capable to express ACE and regulating proteins.

Using this genotype-dependent expression pattern as a tracer, we tested if the primary source of circulating ACE is the lung in humans. To do so, we tested ACE levels in serum and lung samples of the same patients in parallel, using techniques developed in our laboratory in the past years [17,18,20,27,28]. Patients with the DD genotype had significantly higher circulating ACE concentrations and activities than patients with the ACE II genotype, while patients with the ACE ID genotype showed intermediate values confirming earlier reports. However, we did not find any correlation of lung tissue ACE expression or activity with the ACE I/D genotype. This finding suggests that ACE expression in the lungs is independent of ACE I/D genotype, and consequently, the genotype-dependent serum ACE secretion must have an alternative source of ACE. 

The question is, therefore, where do circulating ACE in humans come from? It appears that the lung has the majority of ACE, but it does not contribute proportionally to circulating ACE levels. In accordance, we found a positive correlation between circulating and cardiac (left ventricular) ACE activities. This suggests that secretion of ACE from the human heart significantly contributed to circulating ACE levels. The secretase cleavage site in somatic ACE has been mapped to Arg-1203/Ser-1204 [29]. ADAM17 (also called tumor necrosis factor-α-converting enzyme, TACE) has been proposed as potential secretase [29], but ACE secretion seems to be unaltered in ADAM17/TACE knock out mice, suggesting alternative pathways for ACE secretion [30]. Indeed, one report suggested that the ACE secretase is different from ADAM17/TACE [31]. Nevertheless, there is an apparent consensus in that the mysterious ACE secretase is a membrane-bound enzyme [29,30].

A recent report on lung ACE reported tissue ACE expression decreases in lung cancer [23]. Indeed, a negative correlation between lung cancer and circulating ACE activity was shown half a century ago, in a limited number of patients [32]. This study suggested that if lung microcapillaries are being lost in the tumor, then circulating ACE activities will decline. However, none of the previous studies attempted to directly test the relationships between circulating and lung tissue ACE expressions. To the best of our knowledge, this report is the first to do so in a fairly large human population.

Our data suggest that the source of circulating ACE is independent of lung capillaries. In line with that, the human heart was identified as an alternative source for circulating ACE. Additional ACE expressing and secreting cells can also be found in the apical surface of epithelial cells in the proximal tubule of kidney, the mucosa of small intestine, the syncytial trophoblast of placenta and the choroid plexus, in addition to various regions within the central nervous system [24]. Moreover, ACE was also found to be expressed by macrophages [33]. While the role of these potential ACE sources in the circulating ACE levels is unknown, it is well established that circulating ACE level increases in sarcoidosis [34]. We also confirmed elevated circulating ACE levels in patients with sarcoidosis and proposed that it can be used as a biomarker for sarcoidosis [27,28]. Using a similar approach to ours, an independent study reported genotype-dependent ACE expression in the human heart [15] in full accordance with our findings in the present study, suggestive of a relationship between serum and cardiac ACE activities. 

Another finding of this study is the endogenous regulation of ACE activity by inhibitors. The first results on potential endogenous inhibitors of ACE were reported as early as 1979 [35]. Later human results also suggested the existence of endogenous ACE inhibitors in the heart [36] as well as in the serum by identifying C-type natriuretic peptide [37]. Moreover, it was also shown that dilution can be a valuable tool to investigate the endogenous inhibition of ACE [38], suggesting that ACE is generally inhibited in rat tissues. Our previous reports on the endogenous inhibition of circulating ACE activity [17] by serum albumin [18] were confirmed in the present study. Applying the same technique, we observed a significantly higher endogenous inhibition (approximately 70%) in lung tissue than in blood. These ACE inhibitory levels were comparable in patients with and without ACE inhibitory medications, suggesting a negligible effect of the drug on tissue ACE activities. The concentration of human serum albumin is too low in the lung tissue samples to provide significant ACE inhibition [18], and thus, this implicates an alternative mechanism for ACE inhibition in the present study. These findings were in accordance to that found in the rat, suggesting at least 85% endogenous ACE inhibition in the lung [38]. Further studies are required to identify the molecular nature of the endogenous ACE inhibitor in human lung tissue.

Specific ACE activities were significantly higher in human lung tissues than that in the sera of the same patients. This difference suggests that ACE processing is different in these tissues, resulting in different post-translational modifications. This finding is in accordance with the “conformational fingerprinting” introduced by Danilov et al. [39]. Nonetheless, it is important to note that AngII can also be generated by chymase. Although the human heart exhibits high ACE activity in comparison with other species [25], it appears that chymase predominates over ACE in AngII generation [25,40]. This implies that tissue-localized AngII in the human heart is not determined by the ACE/ACE2 balance, but rather by the chymase/ACE2 balance. On the other hand, ACEi medication is particularly effective in patients with heart failure with reduced ejection fraction (HFrEF), suggesting an important role for ACE in the heart. Unfortunately, chymase activity was not measured in the present study to address this important issue.

We need to acknowledge the limitations of the study. First of all, this is a single-center clinical study, selectively performed on Caucasian patients; therefore, clinical data regarding the correlation between ACEi medication and biochemical efficacy (inhibition) should be considered as exploratory. This study is unbalanced in respect of the results with lung and heart samples: we were unable to provide a side-by-side characterization of lung and heart tissue-localized ACE as a result of limited tissue availability. In particular, we were unable to provide evidence for genotype-dependent ACE expression in the heart or information on the effect of ACEi medication on heart ACE activity.

## Figures and Tables

**Table 1:** General clinical information on the patients enrolled.

Clinical Parameter	End-Stage Heart Failure Patients	Lung Surgery Patients
Enrolled patient’s number	72	108
Age (Median ± IQR)	57.0 ± 11.8	62.6 ± 13.2
Gender (Female/Male)	54/18	37/71
Body mass index (Median ± IQR)	25.99 ± 6.48	N/A
Diabetes (%)	27	10
Ischemic heart disease (%)	38	17
Dilatative heart disease (%)	92	N/A
Left ventricular ejection fraction (Median ± IQR)	57.0 ± 11.75	N/A
Heart failure (%)	100	10
NYHA status (II/III/IV)	3/36/33	N/A
ACE inhibitory therapy (%)	0	51

**Table 2:** There are no effects of age and gender on ACE activities in sera and lungs.

Demographic Parameter	Left Ventricle	Lung
	ACE Activity (U/mg)	ACE Activity (U/mg)
Age < 60 (median ± IQR)	27.6 ± 16.5	114 ± 119
Age ≥ 60 (median ± IQR)	25.8 ± 11.6	117 ± 83
Female (median ± IQR)	29.0 ± 12.2	119 ± 117
Male (median ± IQR)	26.3 ± 14.2	112 ± 93

## Data Availability

The data presented in this study are available on request from the corresponding author. The data are not publicly available due to the clinical information involved.
